# Preclinical models of cardiotoxicity from immune checkpoint inhibitor therapy

**DOI:** 10.1007/s00395-024-01070-0

**Published:** 2024-07-22

**Authors:** Florian Buehning, Tobias Lerchner, Julia Vogel, Ulrike B. Hendgen-Cotta, Matthias Totzeck, Tienush Rassaf, Lars Michel

**Affiliations:** Department of Cardiology and Vascular Medicine, West German Heart and Vascular Center, University Hospital Essen, Hufelandstraße 55, 45147 Essen, Germany

**Keywords:** Cardio-oncology, Cardiotoxicity, Cytotoxic T-lymphocyte-associated protein 4, Immune checkpoint inhibitor, Immune-related adverse events, Programmed cell death protein 1

## Abstract

Immune checkpoint inhibitor (ICI) therapy represents a ground-breaking paradigm in cancer treatment, harnessing the immune system to combat malignancies by targeting checkpoints such as cytotoxic T-lymphocyte-associated protein 4 (CTLA-4) and programmed cell death protein 1 (PD-1). The use of ICI therapy generates distinctive immune-related adverse events (irAEs) including cardiovascular toxicity, necessitating targeted research efforts. This comprehensive review explores preclinical models dedicated to ICI-mediated cardiovascular complications including myocarditis. Tailored preclinical models of ICI-mediated myocardial toxicities highlight the key role of CD8^+^ T cells, emphasizing the profound impact of immune checkpoints on maintaining cardiac integrity. Cytokines and macrophages were identified as possible driving factors in disease progression, and at the same time, initial data on possible cardiac antigens responsible are emerging. The implications of contributing factors including thoracic radiation, autoimmune disorder, and the presence of cancer itself are increasingly understood. Besides myocarditis, mouse models unveiled an accelerated progression of atherosclerosis, adding another layer for a thorough understanding of the diverse processes involving cardiovascular immune checkpoint signalling. This review aims to discuss current preclinical models of ICI cardiotoxicity and their potential for improving enhanced risk assessment and diagnostics, offering potential targets for innovative cardioprotective strategies. Lessons from ICI therapy can drive novel approaches in cardiovascular research, extending insights to areas such as myocardial infarction and heart failure.

## Introduction

In recent years, immune checkpoint inhibitor (ICI) therapy has revolutionized cancer treatment by leveraging the body’s immune system to fight malignancies. In contrast to conventional chemotherapeutics, which in most cases target proliferating properties or specific aberrations of cancer cells, ICIs target immune checkpoints at various stages to induce a specific anti-tumor immune response.

The initiation of an adaptive immune response depends on the crucial step of T cell activation in response to antigen presentation. This activation is triggered by the recognition of antigens by the T cell receptor (TCR). To facilitate this process, multiple costimulatory signals are required. Immune checkpoints play a vital role in counterbalancing T cell activation, preventing an overly exaggerated immune response, and ensuring self-tolerance. Cytotoxic T-lymphocyte-associated protein 4 (CTLA-4) is an immune checkpoint that dampens T cell activation by vying with CD28 for binding to B7 proteins on antigen-presenting cells (APCs). This regulatory process prevents overactive immune responses, preserving immune homeostasis and self-tolerance [75]. Similarly, during the process of T cell activation, programmed cell death protein 1 (PD-1) is expressed and functions to counterbalance positive signals from the TCR and CD28. This is accomplished through interactions with its ligands, programmed cell death 1 ligand 1 and programmed cell death 1 ligand 2 (PD-L1 and PD-L2) [78].

Targeting immune checkpoints has become a promising treatment option for different malignancies. Receiving the first regulatory endorsement in 2011 for the treatment of unresectable or metastatic melanoma, ICIs are now in use for different tumor entities including lung cancer, hepatocellular carcinoma, ovarian cancer, and renal cell carcinoma amongst others. The administration of these medications has resulted in enduring treatment responses and, in some cases, complete remission in patients with advanced-stage cancer [4, 70].

**Fig. 1:**
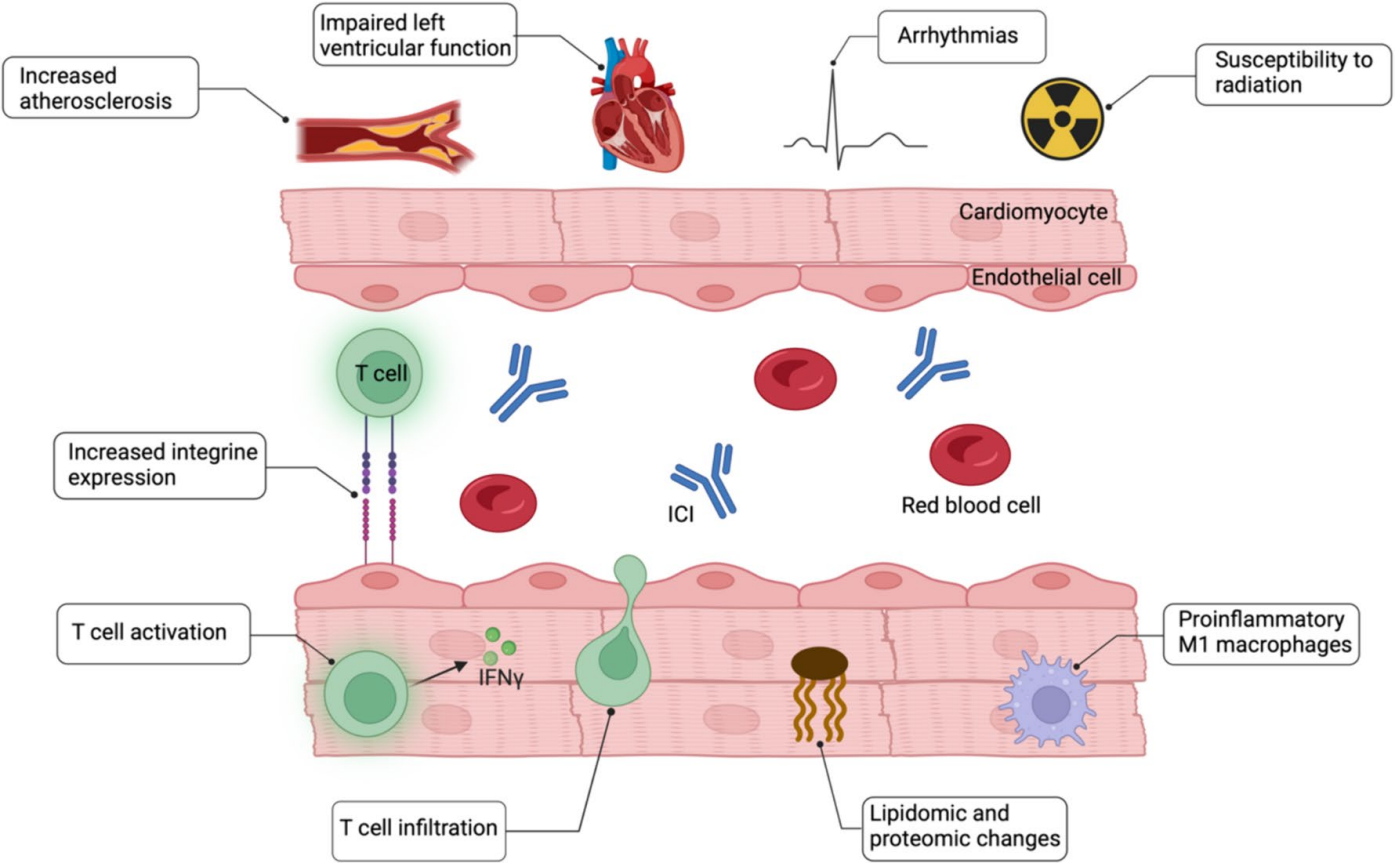
Overview of cellular effects of ICI administration in preclinical models on cardiovascular integrity and functioning. *ICI* immune checkpoint inhibitor. Created with BioRender.com

One downside to these breakthroughs is the onset of a novel range of immune-related adverse events (irAEs), which differ markedly from the traditional toxicities associated with chemotherapy. These irAEs include colitis, pneumonitis, and myocarditis which stands out with the highest fatality rate [53, 59, 84, 85]. Initially considered infrequent and rare, cardiotoxic side effects are now recognized to affect a substantial number of patients undergoing treatment with ICIs, with a 1-year absolute risk of cardiac events reaching up to 9.7% [16]. Nevertheless, variations exist in the incidence of cardiotoxicity-related incidents among ICIs. Typically, ICIs aimed at PD-1 and the combined inhibition of PD-1 and CTLA4 show elevated rates of cardiotoxicity, with the incidence in dual checkpoint inhibition being 87% higher than in monotherapy. Forty percent of patients who developed ICI-induced cardiotoxicity had pre-existing cardiovascular risk factors [43, 76]. The negative impacts mentioned present a notable hurdle to the continued utilization of ICIs. As a result, various preclinical models have been developed to better understand ICI-induced cardiotoxicity, aiming to clarify potential mechanisms for addressing these adverse effects. Three main facets of ICIs within the cardiovascular system are addressed by preclinical models (Fig. 1):

1. *The role of immune checkpoint signaling for basic cardiovascular function.* Broad cardiovascular effects affecting all parts of the cardiovascular system with a diverse phenotype indicate a relevant role of immune checkpoints for cardiovascular function at baseline. The putative involvement of immune checkpoints encompasses preventing overactive immune responses, preserving cardiac immune equilibrium, and exerting distinct effects on myocardial function, metabolic signaling, and metabolic pathways [2, 26, 32, 82].
2. *Predictors and responsible pathways in cardiovascular involvement during ICI therapy.* ICI therapy was shown to induce a wide range of cardiovascular complications, including myocarditis, left ventricular (LV) dysfunction, arrhythmia, and progression of coronary artery disease [5, 37, 48, 60]. A general involvement of key cardiovascular pathways can be demonstrated by various preclinical models, indicating a complex biological phenotype of these complications. Understanding additional risk factors that determine phenotype and severity will be challenging for future research.
3. *Cardiovascular epitopes in ICI-related cardiovascular autoimmune reactions.* Identifying autoantigens that drive ICI-related autoimmune reactions against the cardiovascular system is the focus of current research. Recent data suggest cardiac α-myosin as a potential autoantigen, while another work found evidence for a potential shared epitope between cancer and cardiovascular tissue [8, 43, 86]. Identifying responsible antigens comes with great potential for the development of a targeted cardioprotective approach.

Scientific databases were screened to identify preclinical models of ICI-related cardiotoxicity. Publications until March 2024 are incorporated herein. In total, this review focuses on 15 models for ICI-induced myocardial toxicity described in 19 publications and 10 models for ICI-induced vascular toxicity.

## Myocardial toxicity

**Fig. 2:**
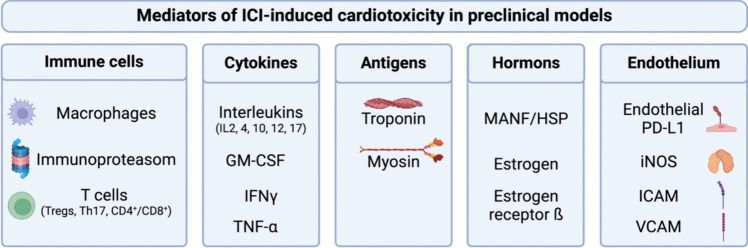
Mediators of ICI-induced cardiotoxicity in preclinical models. *GM-CSF* granulocyte–macrophage colony-stimulating factor, *HSP* heat shock protein, *ICAM* intercellular adhesion molecule 1, *IFNγ* interferon γ, *iNOS* inducible nitric oxide synthase, *MANF* mesencephalic astrocyte-derived neurotrophic factor, *PD-L1* programmed cell death 1, *Tregs* regulatory T cells, *TNF-α* tumor necrosis factor α, *VCAM* vascular cell adhesion molecule. Created with BioRender.com

Occurrence of myocarditis following ICI therapy was initially recorded as case reports and small case series [43]. However, it became apparent that the incidence exceeded initial estimations, and cardiotoxicity other than myocarditis may manifest in a significant number of cases, presenting as LV dysfunction or asymptomatic troponin elevation [13, 52, 57]. Considering these clinical observations, preclinical models are essential to understand the diverse pathophysiology implicated in these heterogeneous presentations of ICI-mediated cardiotoxicity (Fig. 2). Preclinical models for myocardial inflammation designed to study ICI-induced cardiotoxicity will be elaborated in the sections that follow.

## The role of PD-1/PD-L1 in ICI-mediated cardiotoxicity

MRL (Murphy Roths large)/*fas*^*lpr/lpr*^ mice serve as a murine model for a systemic autoimmune syndrome analogous to systemic lupus erythematosus [96]. This model susceptible to autoimmune diseases was used and, by backcrossing with a C57BL/6 background, MRL-*Pdcd1*^*−/−*^ mice were established that developed fatal myocarditis along with infiltration of CD4^+^ and CD8^+^ T cells and myeloid cells. The predominant activity of type 1 T helper (Th) cells and their associated cytokines including interferon γ (IFNγ) was attributed to the observed myocarditis in MRL-*Pdcd1*^*−/−*^ mice. Interestingly, while hepatitis, pneumonitis, gastritis, and sialadenitis were evident, myocarditis was absent in MRL wild-type mice. This underscores the crucial role of PD-1 in regulating the immune response within cardiac tissue. Notably, PD-L1 expression was heightened in cardiomyocytes within inflamed hearts, suggesting a potential mechanism involving the suppression of autoreactive T cells expressing PD-1. The authors identified strong auto-antibody production against cardiac myosin in MRL-*Pdcd1*^*−/−*^ mice. In contrast, MRL mice showed weak antibody production against cardiac myosin prompting that *Pdcd1* deficiency might open the door for the development of myocarditis inclination in MRL mice [86]. Accordingly, *PD-L1*^*−/−;*MRL−*Faslpr*^ mice died earlier than their non-*PD-L1*-deficient controls due to myocarditis-induced heart failure with myocyte destruction orchestrated by macrophages and T cells. The phenotype was not dependent on the *Fas*^*lpr*^ mutation, suggesting that the observed myocarditis and pneumonitis were determinant on the MRL background. The transfer of hematopoietic bone marrow cells was sufficient to induce myocarditis and pneumonitis in wild-type mice. Since cardiac-specific autoantibodies against cardiac troponin I and myosin were shown to increase only after the onset of overt disease, autoantibodies were not considered as key drivers of disease manifestation [50]. Treatment with anti-PD-1 antibodies in A/J mice induced multiorgan inflammation with affection of the heart in 19%. Cardiac T cells showed an activated phenotype, and activation of T cells specific for cardiac myosin was observed. PD-1^+^ cardiac myosin-specific T cells were detectable in naïve mice hinting that ICIs might reactivate those autoreactive T cells leading to ICI-related cardiotoxicity. The administration of interleukin-12 increased the incidence of PD-1 inhibitor-induced myocarditis which was attributed to the enhanced differentiation of pathogenic effector CD8^+^ T cells [32, 91]. Another group identified antibodies against cardiac troponin I as the possible driver for the onset of dilative cardiomyopathy in BALB/c-*Pdcd1*^*−/−*^ mice. The phenotype was not present in BALB/c-*Rag-2*^*−/−*^ mice underlining that the impaired myocardial function in BALB/c-*Pdcd1*^*−/−*^mice is linked to the functions of T and/or B lymphocytes as *Rag-2*^*−/−*^ mice are incapable to produce mature B or T lymphocytes [67, 69, 79]. In an experimental autoimmune-related myocarditis mouse model induced by immunization with murine cardiac troponin I peptide, the immunoproteasome was found to regulate CD4^+^ T cell activation and differentiation towards amplified Th17 and Th1 expansion, while also stimulating proinflammatory cytokines. Deletion of either Low-molecular-weight protein 2/7 as parts of the immunoproteasome or administration of an immunoproteasome inhibitor reduced inflammation and improved cardiac function. Similar inflammatory pathways observed in this murine model have been detected in clinical cases of ICI-related myocarditis, suggesting troponin I as a putative autoantigen [9].

To delineate the specific roles of CD4^+^ and CD8^+^ T cells in the pathogenesis of ICI-related cardiotoxicity, two distinct mouse models of T cell-dependent myocarditis were employed. While the cMy-mOva model of myocarditis relies on CD8^+^ effector T cells, the experimental autoimmune myocarditis (EAM) model in BALB/c mice utilized in research is triggered by CD4^+^ T cells. In both models, the authors effectively illustrated that PD-1 exerts a substantial regulatory role in controlling T cell-mediated inflammation within cardiac tissue [2, 32, 82]. The potential impact of PD-1 on cardiac endothelium was evidenced by the exacerbation of phenotype progression in the acute lymphocytic myocarditis model upon PD-L1 depletion [33].

Further evidence that PD-1/PD-L1 interaction maintains cardiac integrity comes from the mouse model of chronic Chagas disease. Chagas disease is conveyed through the transmission of *Trypanosoma cruzi* (*T. cruzi*), potentially resulting in Chagas cardiomyopathy following the prolonged persistence of the parasitic organisms. In *T. cruzi*-induced acute myocarditis in mice, the introduction of blocking antibodies targeting PD-1 and PD-L1 resulted in an elevated presence of inflammatory infiltrates in the myocardium, accompanied by heightened levels of proinflammatory cytokines such as tumor necrosis factor α (TNF-α) and IFNγ. Remarkably, uninfected C57BL/c-*Pdcd1*^*−/−*^ mice exhibited no indications of myocarditis [35]. Although the combined administration of PD-1/PD-L1 blockade and immunization with irradiated *T. cruzi* led to a decrease in blood parasitemia, it did not affect cytokine production in the heart tissue, contrary to earlier observations [7, 35].

In a different approach, the effects of PD-1 blockade in C57BL/6 mice that underwent targeted radiation of the heart were evaluated. Animals receiving both radiation and PD-1 blockade showed increased mortality, decreased cardiac output along with increased fibrosis and myocardial inflammation. The authors attribute the cardiac toxicities to cytotoxic CD8^+^ T cells as depletion reversed mortality from radiation and PD-1 blockage [17]. Similarly, the application of full thoracic X-ray irradiation to C57BL/6 mice that received anti-PD-1 antibodies increased T cell influx into the heart and lung in comparison to animals that underwent sole irradiation. The combination of irradiation and immunotherapy reduced the 21-day survival rate from 70% (irradiation) to 36% (anti-PD-1 plus irradiation) [64]. This perspective holds significant importance as it clarifies the complexities of combinatorial cancer treatment, offering valuable insights into its practical implementation [61].

Recently, preprint data suggested an increase in CXCL9/10^+^ CCR2^+^ macrophages and emergence of CXCR3hi CD8^+^ T cells in MRL-Pdcd1^−/−^ mice. Bioinformatic analyses indicated an anticipated interaction between these macrophages and T cells. Depletion of macrophages via liposomal clodronate administration resulted in decreased clonal expansion of CD8^+^ T cells in murine hearts and improved overall survival. In MRL mice treated with anti-PD-1 and anti-CTLA-4 antibodies, CXCR3 blockade attenuated immune cell infiltration in the heart, leading to increased survival, particularly by targeting CXCR3hi CD8^+^ T cells. CXCL9/10^+^ macrophages and CXCR3hi CD8^+^ T cells were also found in biopsies from human hearts affected by ICI-myocarditis, suggesting their interaction as a potential novel target for pharmaceutical treatment of ICI-myocarditis [40].

C57Bl/6J mice treated with clinically used doses of pembrolizumab demonstrated early activation of Th17-type cells and mild impairment in systolic function after 1 week. Subsequent intracardial infiltration of T helper cells at 2 weeks exacerbated systolic dysfunction, accompanied by macrophage infiltration, and histologically defined cardiac abnormalities at five weeks. Pembrolizumab administration induced coronary endothelial dysfunction with increased levels of intercellular adhesion molecule 1 (ICAM-1), inducible nitric oxide synthase (iNOS) and significant upregulation of endothelial activation markers like E-selectin. High concentrations of atorvastatin prevented ICI-induced endothelial toxicity in human endothelial cells in vitro and mitigated progressive cardiac dysfunction, cardiac injury, and acute Th17 cytokine storm in the murine model, suggesting its potential as a prophylactic agent against ICI-induced cardiotoxicity [19, 20].

In conclusion, these findings underscore the critical role of PD-1 signaling in maintaining cardiac integrity and regulating immune responses in various models of myocarditis and cardiotoxicity, while inflammatory processes, the interaction of immune cells (e.g. T cells) and immunomodulatory substrates (e.g. TNF-α and IFNγ) present as the driving factors for the induced phenotype.

## The role of CTLA-4 and LAG-3 in ICI-mediated cardiotoxicity

Like PD-1 and PD-L1, CTLA-4 is a key player in preserving peripheral tolerance to the heart. Mice lacking *Ctla4* show a phenotype similar to that of *Pdcd1*-deficient mice, but more severe. They display massive accumulation of T cell blasts in liver, heart, lung, and pancreatic tissue dying by 3–4 weeks of age [83, 87]. Differing from the *Pdcd1* knockout, the phenotype was present in *Ctla4*-deficient mice on both C57BL/6 and BALB/C backgrounds, together with markedly increased levels of IFNγ, interleukin-4 and granulocyte–macrophage colony-stimulating factor (GM-CSF) as an indicator of T cell activation [83]. Studies compared the impact of abrogating CTLA-4 expression in adult mice compared to congenitally *Ctla4*-depleted mice. Whereas multiorgan lymphocyte infiltration was present in both models, the development of myocarditis was unique to mice born with *Ctla4* deficiency. This indicates that autoimmune reactions in mice lacking CTLA-4 display unique organ preferences depending on the specific types of CTLA-4 depletion [45]. In the model of EAM, anti-CTLA-4 treatment of BALB/c mice significantly exacerbated the disease with intense infiltration of lymphocytes into the heart, accompanied by a notable rise in the percentage of CD3^+^ T cells. The authors hypothesized that blocking CTLA-4-B7 interaction enhances Th17-mediated autoimmune responses. This was underlined as the neutralization of IL-17 significantly suppressed the development of the EAM [94]. Another research group corroborated these findings by demonstrating that cardiac function during anti-PD-1 therapy was preserved during specific inhibition of IL-17A. Although minimal myocardial inflammatory changes accompanied by elevated nitrosative stress were evident, an upregulation of thymic cytokine expression highlighted T cell activation within the thymus as a potential mediator of ICI-induced myocarditis. BALB/c mice, characterized by a Th2-dominant immune response, did not display compromised cardiac function. This observation led the authors to propose that discrepancies in systemic immune responses to PD-1 inhibition may predispose individuals to cardiotoxicity development [27].

CD8^+^ T cells were pinpointed as the catalyst for myocarditis in *Pdcd1*^*−/−*^* Ctla4*^+*/−*^ mice. The survival of these mice markedly enhanced following the depletion of CD8^+^ cells, with the removal of CD4^+^ cells showing no substantial impact. Akin to previous reported results, the cardiac-specific protein α-myosin was identified as the origin of the cognate antigen for three major histocompatibility complex class I-restricted TCRs derived from mice undergoing fulminant myocarditis [86]. Taking a translational approach, T cells from the peripheral blood of three patients experiencing ICI-related myocarditis exhibited a robust expansion induced by α-myosin peptides making α-myosin a putative autoantigen in ICI-myocarditis [8].

To further elucidate the role of CTLA-4 on forkhead-Box-Protein P3 (Foxp3^+^) regulatory T cells (Tregs), the influence of specifically deleting the *Ctla4* gene in Foxp3^+^ Tregs in mice on BALB/c background was evaluated. Whereas *Ctla4* knockout mice became moribund at ∼ 20 days of age, *Ctla4* deficiency in Tregs alone showed a less profound phenotype with moribundity after 7 weeks of age. Mice lacking *Ctla4* in Tregs exhibited dense infiltration of mononuclear cells into the myocardium, resulting in the destruction of myocytes and possibly leading to fulminant myocarditis as cause of death. Transferring the splenocytes and purified CD4^+^ T cells into T cell-deficient BALB/c athymic nude mice led to myocarditis, indicating that this condition is T cell-mediated. In both the spleen and lymph nodes, an increased frequency of interleukin-2-, interleukin-4-, and IFNγ-producing Foxp3^+^ CD4^+^ cells were observed. Supporting previous findings, the authors described a rise of IL-17-secreting (Th17) cells in *Ctla4*-deficient mice which was not present in *Ctla4* deficiency in Tregs alone, possibly linking Th17 to accelerated progression to fulminant disease [90, 94]. In a murine transgenic model of CD8^+^ T cell-mediated myocarditis in *Ctla4*^*−/−*^ mice, CD8^+^ T cells dependent on interleukin-12 were identified as a key factor driving inflammation underlining that it is hard to attribute cardiotoxicity of ICIs to one sole interleukin [49].

A transgenic mouse model carrying loss-of-function alleles of *Ctla4* and *Pdcd1* was engineered to explore the effects of T cells and the resulting phenotype in rodents. As documented before, the homozygous loss of *Ctla4* resulted in extensive and lethal lymphoproliferation [83, 87]. In comparison to *Ctla4*^+*/*+^ *Pdcd1*^*−/−*^ mice, *Ctla4*^+*/−*^* Pdcd1*^*−/−*^ mice showed a more pronounced phenotype with lymphocytic infiltration (mainly CD3^+^ CD8^+^ T cells, in a lesser extent CD4^+^ T cells, low amount of Foxp3^+^ Tregs) into the myocardial tissue and myocyte destruction. Whereas no statistically significant differences in echocardiographic parameters were observed, electrocardiographic recordings revealed arrhythmias including sinus node dysfunction, sinus arrest, atrioventricular conduction block, and severe bradycardia in *Ctla4*^+*/−*^* Pdcd1*^*−/−*^ mice. The administration of abatacept (recombinant CTLA-4 Ig) reduced mortality and normalized myocardial immune infiltrates in *Ctla4*^+*/−*^* Pdcd1*^*−/−*^ mice [88].

Sex-related differences in cardiotoxicity are observed in clinical and preclinical data, with females at higher risk [1]. Female C57BL/6J tumor-bearing mice showed more CD8^+^ T cell infiltration and impaired cardiac function after anti-PD-1 and anti-CTLA-4 treatment compared to males. This may be due to lower expression of cardioprotective mesencephalic astrocyte-derived neurotrophic factor and heat shock protein 5 in female mice [28, 29, 97]. Administration of these proteins as recombinant factors attenuated ICI-induced myocarditis. ICI treatment reduced 17-β-estradiol levels in both male and female mice, and activation of estrogen receptor β signaling improved cardiac function and reduced myocardial infiltration by CD8^+^ T cells [98].

Unlike all murine models of ICI-mediated toxicities, a monkey model was established by administering nivolumab (anti-PD-1) and ipilimumab (anti-CTLA-4) to Chinese-origin cynomolgus monkeys. In addition to various organ toxicities, myocarditis was also detected. Transcriptomic analysis revealed heightened migration and activation of T cells, along with increased phagocytosis and antigen presentation within the cardiac tissue. The infiltration of mononuclear cells in the myocardium was primarily comprised of T cells, with fewer macrophages and occasional B cells present. This infiltration was associated with minimal cardiomyocyte degeneration, along with elevated levels of cardiac troponin I and N-terminal pro-B-type natriuretic peptide (NT-pro-BNP) [42].

With the evolution of combinatorial treatment involving various ICIs, there is a pressing necessity to understand the effects of deficiencies in various immune checkpoints on individuals. Initially aiming to introduce a rodent model that is defective in random somatic hypermutation and class switch recombination, a mouse model of spontaneous autoimmune disease was discovered. The autoimmune phenotype in those *Aicda*^*−/−*^ mice was not due to the deficiency in activation-induced cytidine deaminase (Aicda) but rather due to spontaneous loss-of-function mutation in the neighboring gene lymphocyte-activation gene 3 (*Lag3*). *Aicda*^*−/−*^* Pdcd1*^*−/−*^ mice on a BALB/c background died before 10 weeks of age exhibiting dilatation of the heart along with massive lymphocytic infiltration into the atrium and ventricle of the heart. Tregs in BALB/c-*Pdcd1*^*−/−*^* Lag3*^*aida/aida*^ mice had substantial suppressive function, contradicting that myocarditis in this model is mainly orchestrated by the failure of regulatory cells. As *Lag3* deficiency did not induce autoimmunity in non-autoimmune-prone mouse models, the synergistic actions of LAG-3 and PD-1 are crucial in mediating the autoimmunity [68]. Corroborating this, it was demonstrated that *Lag3*^*−/−*^* Pdcd1*^*−/−*^ mice exhibited an early-onset lethal autoimmune condition, manifesting with endocarditis, myocarditis, and pancreatitis [92].

### Early cardiac dysfunction as a manifestation of ICI cardiotoxicity

Our research group was part of the development of a murine melanoma model showing response to anti-PD-1 therapy by transplantation of cutaneous melanoma cells into immunocompetent wild-type C57BL/6N mice. Anti-PD-1 therapy induced a loss of tumor volume along with strong intratumoral recruitment of CD4^+^ and CD8^+^ T cells. Upon administering anti-PD-1 therapy, there was an upregulation of PD-L1 on cardiac endothelial cells. Moreover, notable alterations impacting cellular energy production and equilibrium were detected. Substrates for beta-oxidation significantly increased, while levels of carnitine/acylcarnitine carrier protein, acyl-CoA dehydrogenase, and acyl-CoA synthetase decreased concurrently. Elevated cardiolipin levels were globally observed, suggesting mitochondrial dysfunction after ICI administration. The identified alterations resulted in baseline LV dysfunction and failure to respond to inotropic stress. The removal of CD8^+^ T cells induced the loss of anti-tumor efficacy in anti-PD-1 therapy. In contrast, blocking TNFα reduced cardiotoxicity, as evidenced by maintained LV function alongside preserved anti-tumor response. In mice subjected to anti-PD-1 therapy, the inhibition of TNFα did not impact the augmented recruitment of infiltrating lymphocytes. Still, it resulted in an intensified expression of the immune-inhibitory LAG3, T cell immunoglobulin, and mucin-domain containing-3 (TIM3), indicative of markers associated with lymphocyte exhaustion [56].

Macrophages are abundant within cardiac tissue, and recent research has unveiled their pivotal role in maintaining cardiovascular integrity. In addition to modulating tissue metabolism, they serve as central regulators of immune response within the cardiac microenvironment [24, 46, 66]. Their influence on disease pathology is evident in murine models of chronic heart failure, where the proliferation of proinflammatory macrophages contributes to sustained pathological remodeling [77]. It was demonstrated that the administration of PD-1 inhibitors leads to cardiac function impairment portrayed by LV impairment assessed 28 days after administration of ICIs, accompanied by evident macrophage polarization towards a proinflammatory M1 phenotype both in vivo and in vitro. In C57/Bl6 mice treated with anti-mouse PD-1 inhibitor, proinflammatory macrophage expansion of inducible Nitric Oxygen Synthase (iNOS+) M1 macrophages were observed along with heightened interleukin-1β, interleukin-6, and TNF-α. The restoration of cardiac function, impaired by a PD-1 inhibitor, was achieved by inhibiting MicroRNA-34a (miR-34a), a regulator of macrophage polarization that induces inflammation in cultured macrophages [93].

The previous studies support the idea that treatment involving ICIs induces subclinical alterations in cardiac function, as evidenced by LV dysfunction observed in echocardiography [26]. It is crucial to clarify the factors contributing to the progression of subclinical changes to overt cardiovascular disease in certain instances.

### Dual-hit hypothesis

**Fig. 3:**
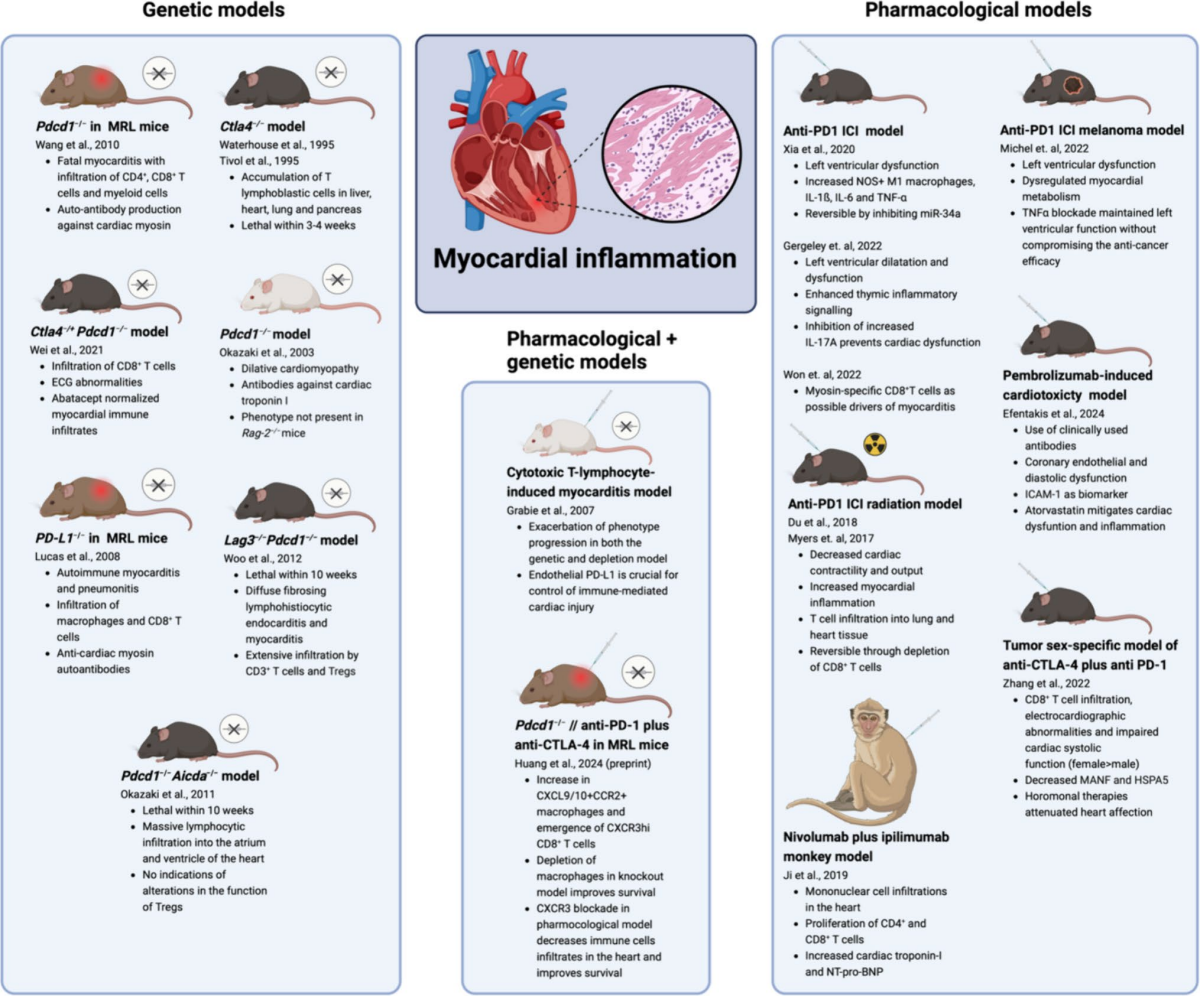
Preclinical models of ICI-mediated cardiotoxicity with different manifestations of cardiac inflammation. *Aicda* activation-induced cytidine deaminase, *Ctla4* cytotoxic T-lymphocyte-associated protein 4 gene, *ECG* electrocardiography, *HSP* heat shock protein, *ICAM-1* intercellular adhesion molecule 1, *ICI* immune checkpoint inhibitor, *IL-1β* interleukin-1β, *IL6* Interleukin-6, *MANF* mesencephalic astrocyte-derived neurotrophic factor, *miR-34a* MicroRNA-34a, *MRL* Murphy Roths large, *NOS* nitric oxygen synthase, *NT-proBNP* N-terminal pro-B-type natriuretic peptide, *Pdcd1* programmed cell death protein 1 gene, *TNF-α* tumor necrosis factor α, *Tregs* regulatory T cells. Created with BioRender.com

Models of myocarditis triggered by ICI therapy commonly embrace a dual-hit hypothesis. These models encompass various properties, including susceptibility to immunological phenomena (MRL and BALB/c mice), myocarditis models (cMy-mOva mice and experimental myocarditis models), infectious disease models (Chagas disease model), radiation-induced models (irradiation in mice), and cancer models (melanoma model) (Fig. 3). Moreover, the dual blockade of multiple immune checkpoints has been shown to enhance the observed effects in these models. Hence, latent alterations at both the molecular and immunological levels induced by ICIs might subsequently evolve into overt cardiovascular toxicity when confronted with an additional predisposing condition or risk factor. Within this framework, the heart could potentially become susceptible to the proinflammatory effects of ICI therapy under any form of cardiac stress. This aligns with the notion of hidden cardiotoxicity, where the toxic effects on the heart from a drug are only apparent in a diseased heart, underscoring the importance of identifying predisposing factors. The dual-hit hypothesis extends the concept of hidden cardiotoxicity by including extracardiac proinflammatory effects as systemic stressors that promote progression from subclinical changes to manifest cardiovascular toxicity [22].

### Atherosclerosis in models for ICI cardiotoxicity

For more than 2 decades, it has been established that atherosclerosis is not the sole accumulation of lipids in arteries; rather, it is an intricate pathophysiological process primarily driven by inflammatory mechanisms [73]. These processes are orchestrated by both the innate and the adaptive immune system and are heavily dependent on the activity of monocytes, macrophages, Th cells, and the release of cytokines [80]. Understanding and eventually throttling the immunologically mediated progression from dysfunctional endothelial lesions to clinically manifest atherosclerosis is a major goal. A meta-analysis highlighted that the development of atherosclerotic-related cardiovascular events in patients under ICI exposure is more likely than in their controls with an odds ratio of 1.51 for myocardial infarction. Therefore, it is crucial to comprehend the pathways of ICI-mediated atherosclerotic effects in preclinical models [14, 15]. To attain this objective, extensively utilized methods include genetic knockout mouse models and pharmacological modulation through ICIs. Studies so far mainly focused on the target proteins CTLA-4, PD-1/PD-L1, and LAG3.

In *Ldlr*^*−/−*^ mice (low-density lipoprotein receptor knockout mouse model) that are commonly used as a model for hypercholesterinemia, short-term anti-CTLA-4/anti-PD-1 treatment did not influence the atherosclerotic plaque area per se. Nonetheless, ICI treatment led to more advanced atherosclerosis plaques with more pathological intima thickening, fibrous cap atheromas, and an increase in plaque necrotic core area. This went along with more CD8^+^ T cell infiltration possibly provoking an increase in necrotic core size of atherosclerotic plaques through macrophage death. The elevated presence of adhesion molecules such as vascular cell adhesion molecule 1 (VCAM-1) and ICAM-1 on the arterial endothelium might have facilitated the infiltration of T cells into the arterial wall of mice treated with ICIs [71]. The same research team also investigated how the antibody-mediated inhibition of CTLA-4 aggravates atherosclerotic plaque inflammation in *Ldlr*^*−/−*^ mice. CTLA-4 treatment induced an activated T cell profile and endothelial activation. Atherosclerotic plaque area was found to be twice as large with a more advanced morphological phenotype and an increased T cell/macrophage ratio [72].

The overexpression of CTLA-4 in apolipoprotein E-deficient mice ((CTLA-4-Tg)/Apoe(-/-)) resulted in diminished formation of atherosclerotic lesions and reduced intraplaque accumulation of macrophages and CD4^+^ T cells in the aortic root. This went along with a decreased expression of the costimulatory molecules CD80 and CD86 acting as ligands for CTLA-4 as well as the costimulatory molecule CD28 on dendritic cells [54]. Blockage of this costimulatory CD-28-CD80/86-T cell activation through abatacept-treatment decelerated atherosclerosis in ApoE3*Leiden mice acting as a common preclinical model of accelerated atherosclerosis [21]. Likewise, in a model of hyperhomocysteinemia (HHcy)-accelerated plaques in apolipoprotein E-deficient (*apoE*^*–/–*^) mice abatacept-treatment reversed plaque development with reduced T cell-dependent macrophage recruitment and IFN-γ/Interleukin-2 secretion [51]. Similar to those discoveries, it was demonstrated that *Pdl1*^*−/−*^* Ldlr*^*−/−*^ mice developed larger atherosclerotic lesions compared to *Ldlr*^*−/−*^ mice. More abundant CD4^+^ and CD8^+^ T cells and macrophages were part of those lesions [10]. Similarly, after 10 weeks of high-cholesterol diet feeding, the intima of *Pdl1/2*^*–/–*^*LdlrR*^*–/–*^ mice showed a threefold increase in CD4^+^ T cells as well as a marked increase in CD8^+^ T cell infiltration in the intima. This coincided with a general rise in the atherosclerotic burden observed in the aortic sinus and arch of these animals.

The PD-1/PD-L pathway is believed to limit atherosclerosis by downregulating proatherogenic T cell response and limiting APC-dependent T cell activation [31]. Echoing this hypothesis and underscoring the distinctive impact on specific T cell populations, the stimulation of the PD-1 pathway in *Ldlr*^*−/−*^ mice led to a decrease in atherogenic IFNγ-producing splenic CD4^+^ T cells, whereas atheroprotective IL-10-producing CD4^+^ T cells were elevated. Overall, the diminished immune activation following the administration of agonistic PD-1 antibodies reduced atherosclerosis development [34]. In reperfused acute myocardial infarction (repAMI), anti-PD-1 therapy fosters the infiltration of CD8^+^ T cells into the myocardium, without influencing the sizes of the infarct. There was no significant alteration observed in the count of B cells and CD4^+^ T cells. Nonetheless, these findings underline the immediate effects of ICIs on cardiovascular integrity [58].

The effects of ICI therapy seem to be diverse and involve different immunological pathways as it was outlined that interrupting PD-1 signaling boosts not only the widespread activation of proatherogenic IFN- γ releasing Th1 cells but also of atheroprotective Foxp3^+^ Tregs. However, the authors showed in their studies using *Ldlr*^*−/−*^*Pdcd1*^*−/−*^ mice that the proatherogenic effects prevail as shown by enhanced dyslipidemia, vascular inflammation, and atherosclerosis [11].

The immune checkpoint receptor LAG3 is the most recent target for immune checkpoint therapy, with recent FDA approval for antibody treatment [63]. LAG3 is expressed on different subsets of leucocytes and mainly acts through interaction with its ligands MHC Class II, Galectin-3, and LSECtin. Although not yet fully understood LAG3 regulates T cell function and expansion [6, 41, 47]. Murine hypercholesterolemic models of atherosclerosis were utilized to investigate the role of LAG3 in the disease process. While not promoting atherosclerotic plaque size, both *Lag3*-deficient and anti-LAG3 treated mice showed enhanced density of T cells in plaques compared to controls. Further, levels of IFN-γ-producing Th1 cells, effector/memory T cells, and regulatory T cells were increased. In particular, the combinatorial blockage of PD-1 and LAG3 increased those effects compared to the mono-blockage of LAG3 [62].

The existing preclinical evidence emphasizes the potential hazard posed by immune checkpoint inhibitor-induced exaggerated atherosclerosis. The available evidence indicates that the proatherosclerotic effects primarily stem from heightened T cell-modulated inflammation within atherosclerotic plaques after ICI administration (Table 1). A more profound comprehension of these mechanisms holds the promise of mitigating the impact on atherosclerotic progression following ICI treatment and concurrently advancing our insights into the broader therapeutic landscape for atherosclerotic diseases. Thus far, preclinical research has pinpointed abatacept as a potential medication alleviating the proatherogenic impacts of ICIs. Observational studies indicate that the proatherosclerotic effects of ICIs might be modulable by statins. However, whether convenient drugs used in cardiovascular disease (CD) are safe to use and efficient in a collective of non-CD patients remains unknown [15]. In a multicenter observational retrospective study, the use of β-blockers, aspirin, and statins was independently related to an increased objective response rate in patients treated with ICIs favoring the use of those drugs [12]. Prospective clinical studies and preclinical models are needed to further support these correlations.

**Table 1:** Preclinical models of the impact of immune checkpoints and their pharmacological modulation on the development of atherosclerosis

Target	Model	Effects on phenotype and molecular mechanisms
PD-1/PD-L1	*Ldlr*^*−/−*^ mice with western-type diet plus agonistic PD-1 antibody [34]	Reduced T cell activation and proliferation Less CD4^+^ and CD8^+^ T cell in atherosclerotic plaques Increased atheroprotective IL-10-producing CD4^+^ T cells Reduced atherosclerotic burden
	*Pdcd1*^*−/−*^ mice with in vivo repAMI [58]	Increased numbers of CD8^+^ T cells in repAMI No alterations in infarction size
	*Ldlr*^*−/−*^ mice and cholesterol diet plus ICI or in *Pdcd1*^*−/−*^ mice [10]	Larger atherosclerotic lesions More abundant CD4^+^ and CD8^+^ T cells and macrophages Elevated serum TNF-α levels More cytotoxic activity of CD8^+^ T cells
	*Pdl1/2*^*−/−*^*Ldlr*^*−/−*^ mice with cholesterol-enriched diet [31]	Increased atherosclerotic burden with more lesional CD4^+^ and CD8^+^ T cells Elevated serum TNF-α levels Enhanced APC-dependent T cell activation
	*Ldlr*^*−/−*^* Pdcd1*^*−/−*^ and high fat diet [11]	Increased systemic CD4^+^ and CD8^+^ T cell activation Expansion of proatherogenic IFNγ-secreting Th cells and atheroprotective Foxp3^+^ Tregs T cell infiltration in atherosclerotic lesions and exacerbated atherosclerotic lesions Increased hypercholesterolemia
CTLA-4 and PD-1	*Ldlr*^*–/–*^ mice on a 0.15% cholesterol diet plus anti-CTLA-4 and anti-PD-1 antibodies [71]	Activated T cell profile No effect on plaque area per se More advanced atherosclerosis with increased plaque necrotic core Endothelial activation with increased expression of ICAM-1 and VCAM-1
CTLA-4	*Ldlr*^*–/–*^ mice on a 0.15% cholesterol diet plus anti-CTLA-4 [72]	Activated T cell profile Plaques with more advanced morphological phenotype and an increased T cell/macrophage ratio Smooth muscle cell and collagen content decreased in plaques
	Overexpression of CTLA-4 in apolipoprotein E-deficient mice [54]	Reduced atherosclerotic lesions and intraplaque accumulation of macrophage and CD4^+^ T cells Decreased proliferative capacity of CD4^+^ T cells and their proinflammatory cytokine production Decreased expression of costimulatory molecules CD80 and CD86
	Hypercholesterolemic ApoE3*Leiden with abatacept-treatment [21]	Reduced accelerated atherosclerosis development Less CD4^+^ T cell activation Elevated IL-10 and decreased IFNγ plasma levels
	Apolipoprotein E-deficient mice with HHcy-accelerated atherosclerosis plus abatacept-treatment [51]	Reverse of accelerated atherosclerosis Less augmentation of CTLA-4 endocytosis caused by Hcy and release of inflammatory cytokines in T cells
LAG3	*Ldlr*^*−/−*^ mice and high-cholesterol diet plus ICI or in *Lag3*^*−/−*^ mice. [62]	Increased levels of IFNγ-producing Th1 cells and effector/memory T cells Increased levels of regulatory T cells The density of T cells within plaques doubled No affection of plaque size

## Translating preclinical models to understand ICI-induced cardiotoxicity

Several studies highlighted the similarities between preclinical and clinical findings regarding ICI-mediated cardiotoxicity. Case reports of patients presenting with myocarditis under ICI treatment have shown the infiltration of T cells and macrophages in myocardial tissue [25, 43]. As preclinical models outlined before, overall proinflammatory processes mediated by cytokines and activation of Th17 cells were clinically observed as driving factors in mediating inflammatory processes during ICI treatment, leading to LV dysfunction [18, 23, 44, 56, 74, 89]. Translational research approaches have identified both myosin and troponin as putative autoantigens in the mediation of ICI-induced myocarditis [8, 9, 43]. Autopsies comparing cancer patients who underwent ICI treatment and those who did not receive ICIs showed an altered inflammatory cell composition in coronary artery atherosclerotic plaques. The inflammatory response was predominantly T cell-driven, in line with preclinical findings in Ldlr^−/−^ mice. Given the protracted progression and multifaceted etiology of atherosclerosis, clinical data are necessary to understand the true phenotype [65, 71]. Incorporating cardioprotective strategies like remote ischemic preconditioning, into translational research is essential for a comprehensive understanding [38, 39].

## Limitations of preclinical models of ICI-mediated cardiotoxicity

The preclinical models used to assess ICI-induced cardiotoxicity described in this review exhibit various limitations. Knockout models, which eliminate pharmacokinetics of ICIs may poorly mirror the clinical use of disease [55]. Approaches based on the administration of ICIs use murine-specific antibodies at doses that exceed those in clinical practice which may lead to an overestimation or insufficient representation of the effects, thus limiting transfer to patients [3, 27, 30, 93].

The impact of the microbiome and environmental pathogens on the immune system is considered highly important in mediating ICI-related anti-cancer effects, but is inadequately represented in animal models of immune-mediated diseases [36, 81]. Mouse models do not sufficiently cover human heterogeneity, including age, gender, social factors, comorbidities or race [1, 88, 95].

## Conclusion

Preclinical models of ICI-mediated cardiotoxicity play a crucial role in understanding and addressing potential cardiovascular side effects associated with this revolutionary cancer therapy. These models often exhibit a predisposing condition together with immune checkpoint blockade or deficiency, suggesting that pre-existing vulnerabilities contribute to cardiac adverse events. CD8^+^ T cells emerge as the primary contributors to cardiac inflammation following ICI administration. The involvement of other immune cells, such as CD4^+^ T cells, B cells, monocytes, and Tregs exhibits inconsistency across various studies. Troponin and α-myosin are recognized as potential autoantigens implicated in ICI-related cardiotoxicity but a clinical transfer is missing so far. Preclinical models not only contribute to the comprehension of ICI cardiotoxicity and the exploration of potential therapeutic interventions for managing associated side effects but also offer insights into the influence of immune checkpoints on cardiac integrity in healthy individuals. They provide valuable information regarding the implications of immune checkpoint activity on the progression of cardiac diseases. Future investigations must adopt both *bench-to-bedside* and *bedside-to-bench* approaches to bridge the gap between preclinical models and the human pathophysiology of ICI-induced cardiotoxicity. A systematic assessment of cardiovascular complications in oncological studies is crucial for the early identification of complications that must be targeted in future translational research (*bedside-to-bench*). A close interface with clinical data will enhance the transferability of preclinical findings to the clinic (*bench-to-bedside*), facilitated using adequate experimental models. These strategies aim to advance diagnostics, therapeutics, and especially preventive measures against ICI-induced cardiotoxicity.

## Data Availability

All sources and datasets are publicly available and cited within the article.
